# Next‐generation sequencing improves molecular epidemiological characterization of thalassemia in Chenzhou Region, P.R. China

**DOI:** 10.1002/jcla.22845

**Published:** 2019-02-27

**Authors:** Haoqing Zhang, Caiyun Li, Jianbiao Li, Shuai Hou, Danjing Chen, Haiying Yan, Shiping Chen, Saijun Liu, Zhenzhen Yin, Xiaoqin Yang, Jufang Tan, Xiaoyan Huang, Liming Zhang, Junbin Fang, Caifen Zhang, Wei Li, Jian Guo, Dongzhu Lei

**Affiliations:** ^1^ Center of Prenatal Diagnosis Chenzhou No. 1 People’s Hospital Chenzhou China; ^2^ BGI‐Shenzhen Shenzhen China; ^3^ China National GeneBank BGI‐Shenzhen Shenzhen China; ^4^ BGI Genomics, BGI‐Shenzhen Shenzhen China; ^5^ Clinical Laboratory of BGI Health BGI‐Shenzhen Shenzhen China

**Keywords:** mutation, next‐generation sequencing, prevalence, thalassemia, variant

## Abstract

**Objectives:**

Thalassemia is a highly prevalent monogenic inherited disease in southern China. It is important to collect epidemiological data comprehensively for proper prevention and treatment.

**Methods:**

In this study, blood samples collected from 15 807 residents of Chenzhou were primarily screened by hematological tests. A total of 3973 samples of suspected thalassemia carriers were further characterized by combined next‐generation sequencing (NGS) and Gap‐PCR.

**Results:**

In total, 1704 subjects were diagnosed as thalassemia carriers with a total prevalence rate of 10.78%, including 943 α‐thalassemia carriers, 708 β‐thalassemia carriers, and 53 composite α and β‐thalassemia carriers. The prevalence rates of α‐thalassemia, β‐thalassemia, and composite α and β‐thalassemia were 5.97%, 4.48%, and 0.34%, respectively. Meanwhile, we characterized 19 α‐thalassemia variations and 21 β‐thalassemia variations in thalassemia carriers. Approximately 2.88% of thalassemia carriers would be missed by traditional genetic analysis. In addition, four novel thalassemia mutations and one novel abnormal hemoglobin mutation were identified.

**Conclusions:**

Our data suggest a high prevalence of thalassemia and a diverse spectrum of thalassemia‐associated variations in Chenzhou. Also, combined NGS and Gap‐PCR is an effective thalassemia screening method. Our findings might be helpful for prevention and treatment of thalassemia in this region.

## 1 INTRODUCTION

Thalassemia is one of the most prevalent monogenic inherited disorders in the world. Approximately 5% of the population worldwide are thalassemia carriers. Due to population growth in recent decades, the number of births suffered from thalassemia is increasing, especially in developing and low‐income regions.^1^, ^2^

Thalassemia is characterized by reduced or even absent production of one of the subunits of hemoglobin. The majority of adult hemoglobin is composed of two α‐globin and two β‐globin subunits, while fetal hemoglobin is composed of two α‐globin and two γ‐globin subunits. Thalassemia mainly consists of α and β‐thalassemia. For α‐thalassemia, because of the absence or reduced production of α‐globin chains, excess β chains or γ chains form non‐functional tetramers, which are called hemoglobin H and hemoglobin Bart's, respectively. Hemoglobin H could form inclusion bodies which are harmful to erythrocytes. On the contrary, β‐thalassemia is caused by little or reduced production of β‐globin chains. Hence, erythrocytes would be damaged by insoluble aggregates formed by excess free α‐globin chains.^1^, ^2^, ^4^

Clinically, thalassemia has variable manifestations ranging from absence of symptoms to fatal. Thalassemia is mainly classified as thalassemia trait, thalassemia intermedia, and thalassemia major according to clinical severity. The latter two subgroups are also diagnosed as thalassemia patients. The phenotypic severity of the disease mainly correlates with degree of imbalance of α:non‐α chains.^5^

Although prognosis for thalassemia has been markedly improved, lifelong care is required for many cases.^6^, ^7^ Proper treatment brings substantial financial burden to patients as well as society in prevalent areas.^11^ Accordingly, prevention of births with thalassemia is particularly important. Comprehensive molecular epidemiological data of the disease are necessary for proper prevention and treatment. At present, combined reverse dot blot (RDB) and Gap‐PCR is the most commonly used method in identifying thalassemia mutations.^12^ The major limitation of these methods is that only common variations could be identified. Therefore, it is required to develop novel technology to screen mutations comprehensively. Recently, next‐generation sequencing (NGS) was used to screen thalassemia carriers in a few studies of China.^13^, ^14^ These studies indicated that the spectrum of thalassemia‐associated variations was much broader than previously reported and suggested that NGS was an effective method in screening thalassemia‐associated variations to facilitate diagnosis.

Thalassemia is popular in tropical and subtropical regions, including South China. Chenzhou is the southernmost city of Hunan Province, People's Republic of China, and sits on the border of Hunan and Guangdong provinces. In China, Guangdong and Guangxi provinces have the highest prevalence of thalassemia. Our previous results showed a high prevalence rate of thalassemia in Chenzhou by RDB and Gap‐PCR.^16^ However, the spectrum of thalassemia variations was not comprehensive. We speculated that many types of thalassemia variations could be missed. Here, we firstly combined NGS and Gap‐PCR in screening thalassemia variations in Chenzhou Region to assess thalassemia variation burden comprehensively and its potential application in preventing births with thalassemia.

## 2 MATERIALS AND METHODS

### 2.1 Participants

This study was approved by BGI's institutional review board on bioethics and biosafety and the Ethic Committee of Chenzhou No. 1 People's Hospital. Written informed consents of all participants were obtained. A total of 15 807 participants (6164 men and 9643 women), who visited hospital for routine medical examination between April 2015 and February 2017, were enrolled in this study. Participants with following cases were excluded from the study: (a) incomplete information, (b) consanguinity, and (c) lack of informed consent. The age distribution of the participants was as follows: 1‐15 years old, 2901; 16‐25 years old, 3390; 26‐35 years old, 6819; 36‐45 years old, 2540; and 46‐53 years old, 157. The family members of novel thalassemia mutation carriers were directly screened by hematological tests and genetic analysis and were not included in this cohort.

### 2.2 Primary hematological screening

Peripheral venous blood samples were obtained from all participants. All samples were primarily screened with routine blood examination and/or hemoglobin electrophoresis. Subjects were considered as suspected thalassemia carriers if either of the following parameters was tested positive: (a) mean corpuscular volume (MCV) <82 fl and/or mean corpuscular hemoglobin (MCH) <27 pg, (b) Hb A2 concentration < 2.5% and Hb F concentration < 2%, and (c) Hb A2 concentration > 3.5% and Hb F concentration at >3.5%. Suspected thalassemia subjects were subjected to further genetic analysis.

Routine blood examinations were performed with ADVIA 2120i Hematology System (Siemens Healthineers, Erlangen, Germany). Hemoglobin electrophoresis was performed with Capillarys 2 Flex Piercing (SEBIA, Lisses, France).

### 2.3 DNA extraction

Genomic DNA of suspected thalassemia carriers was extracted from whole blood using QIAamp DNA Blood Mini Kit (Qiagen, Hilden, Germany). The concentration of DNA samples was quantified by Qubit 3.0 Fluorometer (Thermo Fisher Scientific, Waltham, MA, USA).

### 2.4 Gap‐PCR

The three most common α‐thalassemia‐associated deletions (‐‐^SEA^, ‐α^3.7^, and ‐α^4.2^) and two rare deletions (‐‐^FIL^ and ‐‐^THAI^) in China were characterized by the Gap‐PCR. Two common β‐globin gene deletions (SEA‐HPFH and Gγ^+^(^As^γδβ)) were also analyzed by Gap‐PCR.

### 2.5 NGS screening

The full length of *HBA1*, *HBA2*, and *HBB* was amplified by PCR. The amplicons spanned all the exons and introns of *HBA1*, *HBA2*, and *HBB* genes, which ensured that most thalassemia‐associated mutations and CNVs in the HbVar database could be detected. Sequencing libraries were constructed according the Illumina HiSeq sequencing library preparation protocol. These libraries were further paired‐end‐sequenced for 100 base pairs (PE100) with an Illumina HiSeq 2000 machine. The protocol of bioinformatic analysis of identifying hemoglobin gene variations was described previously.^14^ All variations were validated with Sanger sequencing.

## 3 RESULTS

### 3.1 Thalassemia carriers found by NGS

In total, 15 807 subjects were primarily screened by hematological examinations and 3973 suspected subjects were further analyzed by NGS and Gap‐PCR. Among these subjects, 1704 subjects were diagnosed as thalassemia carriers, including 943 α‐thalassemia carriers, 708 β‐thalassemia carriers, and 53 composite α and β‐thalassemia carriers. The overall prevalence rate of thalassemia in Chenzhou was 10.78%, and the prevalence rates of α and β‐thalassemia were 5.97% and 4.48%, respectively. In addition, the rate of composite α and β‐thalassemia was firstly determined in Chenzhou, which was found in 0.34% of all subjects.

Among 996 carriers with α‐thalassemia variations, we identified 19 different variations with 30 distinct genotypes in this study (Table 1). ‐‐^SEA^/αα was the most abundant α‐thalassemia genotype with a proportion of 67.87%. ‐α^3.7^/αα, ‐α^4.2^/αα, and ‐α^3.7^/‐‐^SEA^ genotypes occurred frequently and represented 14.86%, 4.42%, and 3.41% of all genotypes, respectively. Notably, rare variations such as HBA2:c.95+5_95+28delGGCTCCCTCCCCTGCTCCGACCCG, initiation codon (‐T), and IVS‐I‐117 (G>A) were firstly reported in Mainland China. In addition, two novel α‐globin mutations, including HBA2:c.6_7insTG and HBA1:c.2T>C, were discovered in three unrelated individuals.

**Table 1:** Distribution of α‐thalassemia genotypes in Chenzhou Region

Genotype	Number	Frequency (%)
‐‐^SEA^/αα	676	67.87
‐α^3.7^/αα	148	14.86
‐α^4.2^//αα	44	4.42
‐α^3.7^/‐‐^SEA^	34	3.41
α^CS^α/αα	17	1.71
α^WS^α/αα	15	1.51
α^QS^α/αα	9	0.90
Alpha2 Codon 30 del GAG/αα	8	0.80
α^CS^α/‐‐^SEA^	8	0.80
‐α^4.2^/‐‐^SEA^	6	0.60
‐α^3.7^/‐α^3.7^	3	0.30
Initiation codon (‐T)/αα	3	0.30
‐‐^THAI^/αα	3	0.30
Alpha2 Codon 31 (AGG>AAG)/αα	2	0.20
α^WS^α/‐‐^SEA^	2	0.20
HBA2:c.46G>A(Gly>Ser)/αα	2	0.20
HBA2:c.6_7insTG/αα	2	0.20
HBA2:c.95+5_95+28delGGCTCCCTCCCCTGCTCCGACCCG/αα	2	0.20
‐α^3.7^/‐α^4.2^	1	0.10
‐α^4.2^/‐α^4.2^	1	0.10
Codon 116 (G>T)/αα	1	0.10
α^CS^α/‐α^3.7^	1	0.10
α^QS^α/‐‐^SEA^	1	0.10
HBA1:c.2T>C/αα	1	0.10
HBA1:c.429+51_429+53delCCT/αα	1	0.10
HBA2:c.184A>T(Lys>End)/αα	1	0.10
IVS‐I‐117 (G>A)/‐‐^SEA^	1	0.10
ααα^3.7^/‐‐^SEA^	1	0.10
HBA2:c.46G>A(Gly>Ser)/‐‐^SEA^	1	0.10
HBA2:c.95+5_95+28delGGCTCCCTCCCCTGCTCCGACCCG/‐α^3.7^	1	0.10
Total	996	100.00

In this cohort, we also found 21 β‐thalassemia mutations and 32 genotypes in 761 subjects (Table 2). Codons 41/42 (‐TTCT)/β^N^ and IVS‐II‐654 (C>T)/β^N^ are the top two most frequent genotypes. The ranking order of the two major genotypes is distinct from our previous result. The proportions of Codons 41/42 (‐TTCT)/β^N^ and IVS‐II‐654 (C>T)/β^N^ were 34.69% and 30.62%, respectively, in the current study. However, in our previous study, the proportions of Codons 41/42 (‐TTCT)/β^N^ and IVS‐II‐654 (C>T)/β^N^ were 31.00% and 37.99%, respectively.^16^ The remaining common genotypes were Codon 17 (A>T)/β^N^, −28 (A>G)/β^N^, and Codons 71/72 (+A)/β^N^ with corresponding proportions of 12.61%, 6.70%, and 4.20%. Meanwhile, rare β‐thalassemia mutation IVS II‐761 A>G was identified for the first time in Mainland China. Moreover, we also characterized two novel β‐thalassemia mutations, including HBB:c.260 or 261delC and HBB:c.43delC.

**Table 2:** Distribution of β‐thalassemia genotypes in Chenzhou Region

Genotype	Number	Frequency (%)
Codons 41/42 (‐TTCT)	264	34.69
IVS‐II‐654 (C>T)	233	30.62
Codon 17 (A>T)	96	12.61
‐28 (A>G)	51	6.70
Codons 71/72 (+A)	32	4.20
Hb E	14	1.84
Codons 27/28 (+C)	11	1.45
Codon 43 (G>T)	11	1.45
‐29 (A>G)	10	1.31
‐50 G>A	7	0.92
Codons 14/15 (+G)	5	0.66
Codons 41/42 (‐TTCT)/Codons 41/42 (‐TTCT)	3	0.39
‐90 (C>T)	2	0.26
5'UTR +43 to +40 (‐AAAC)	2	0.26
CD37 (TGG>TAG)	2	0.26
IVS‐I‐1 (G>T)	2	0.26
Codon 17 (A>T)/‐28 (A>G)	1	0.13
Codons 41/42 (‐TTCT)/‐28 (A>G)	1	0.13
Codons 41/42 (‐TTCT)/‐50 G>A	1	0.13
Codons 41/42 (‐TTCT)/Hb E	1	0.13
Codons 41/42 (‐TTCT)/IVS‐II‐654 (C>T)	1	0.13
Codons 41/42 (‐TTCT)/SEA‐HPFH	1	0.13
IVS‐II‐654 (C>T)/‐28 (A>G)	1	0.13
IVS‐II‐654 (C>T)/IVS‐II‐654 (C>T)	1	0.13
IVS‐II‐654 (C>T)/Hb E	1	0.13
‐31 (A>C)	1	0.13
Hb E/Hb E	1	0.13
HBB:c.260 or 261delC	1	0.13
HBB:c.43delC	1	0.13
Initiation codon ATG>AGG	1	0.13
IVS II‐761 A>G	1	0.13
SEA‐HPFH	1	0.13
Total	761	100.00

Fifty‐three subjects were carriers with both α‐ and β‐globin variations (Table 3). Among these carriers, 83.02% of genotypes consisted of common deletions of α‐globin gene (αα/‐‐^SEA^, αα/‐α^3.7^, αα/‐α^4.2^) combined with a β‐globin gene point mutation. Among these genotypes, composite αα/‐‐^SEA^ and Codons 41/42 (‐TTCT)/β^N^ was the most frequent genotype.

**Table 3:** Genotypes of composite α and β‐thalassemia in Chenzhou Region

α	β	Number
‐‐^SEA^/αα	Codons 41/42 (‐TTCT)/β^N^	9
‐‐^SEA^/αα	‐28 (A‐>G)/β^N^	5
‐‐^SEA^/αα	‐50 G>A/β^N^	4
‐‐^SEA^/αα	IVS‐II‐654 (C‐>T)/β^N^	4
‐α^3.7^/αα	IVS‐II‐654 (C‐>T)/β^N^	4
‐α^3.7^/αα	Codons 41/42 (‐TTCT)/β^N^	3
‐α^4.2^/αα	IVS‐II‐654 (C‐>T)/β^N^	3
‐α^3.7^/αα	Hb E/β^N^	2
‐α^4.2^/αα	Codons 41/42 (‐TTCT)/β^N^	2
‐‐^SEA^/αα	Codon 17 (A‐>T)/β^N^	1
‐‐^SEA^/αα	Codons 14/15 (+G)/β^N^	1
‐‐^SEA^/αα	Codons 27/28 (+C)/β^N^	1
‐α^3.7^/αα	‐29 (A‐>G)/β^N^	1
‐α^3.7^/αα	5'UTR +43 to +40 (‐AAAC)/β^N^	1
‐α^3.7^/αα	Codon 17 (A‐>T)/β^N^	1
‐α^3.7^/αα	Codon 43 (G‐>T)/β^N^	1
‐α^4.2^/αα	‐28 (A‐>G)/β^N^	1
‐α^3.7^/‐‐^SEA^	IVS‐II‐654 (C‐>T)/β^N^	1
α^WS^α/αα	Codon 43 (G‐>T)/β^N^	1
α^WS^α/αα	Codons 41/42 (‐TTCT)/β^N^	1
HBA2:c.46G>A(Gly>Ser)/αα	Codons 41/42 (‐TTCT)/β^N^	1
HBA2:c.46G>A(Gly>Ser)/αα	IVS‐II‐654 (C‐>T)/β^N^	1
Initiation codon (‐T)/αα	‐28 (A‐>G)/Codon 17 (A‐>T)	1
Alpha2 Codon 30 del GAG/αα	Codon 17 (A‐>T)/β^N^	1
Alpha2 Codon 31 (AGG>AAG)/αα	IVS‐II‐654 (C‐>T)/β^N^	1
HBA1:c.429+51_429+53delCCT/αα	Codons 71/72 (+A)/β^N^	1

In addition, 13 abnormal hemoglobin variants were identified in 35 subjects with a carrier rate of 0.22% (Table 4). Among these subjects, nine subjects and five subjects were simultaneously affected by α‐thalassemia mutations and β‐thalassemia mutations, respectively. Three rare abnormal hemoglobin variants, Hb Zurich‐Langstrasse, Hb Yusa, and Hb Genova, were reported for the first time in China. Moreover, we found a new case of Hb Savaria with a novel mutation HBA2:c.150C>G, which was predicted as one of the three mutated forms of Hb Savaria before.^17^

**Table 4:** Abnormal hemoglobin variants in this cohort

Abnormal hemoglobin variant	Number	Frequency (%)
Hb New York	8	0.0454
Hb Hekinan II	7	0.0397
Hb Q‐Thailand	4	0.0227
Hb Zurich‐Langstrasse	4	0.0227
Hb G‐Taipei	2	0.0113
Hb J‐Bangkok	2	0.0113
Hb Port Phillip	2	0.0113
Hb Zurich‐Albisrieden	1	0.0057
Hb Genova	1	0.0057
Hb G‐Honolulu	1	0.0057
Hb Hamilton	1	0.0057
Hb Yusa	1	0.0057
Hb Savaria (HBA2:c.150C>G(Ser>Arg))	1	0.0057
Total	35	0.1985

### 3.2 Characterization of novel mutations

**Figure 1:**
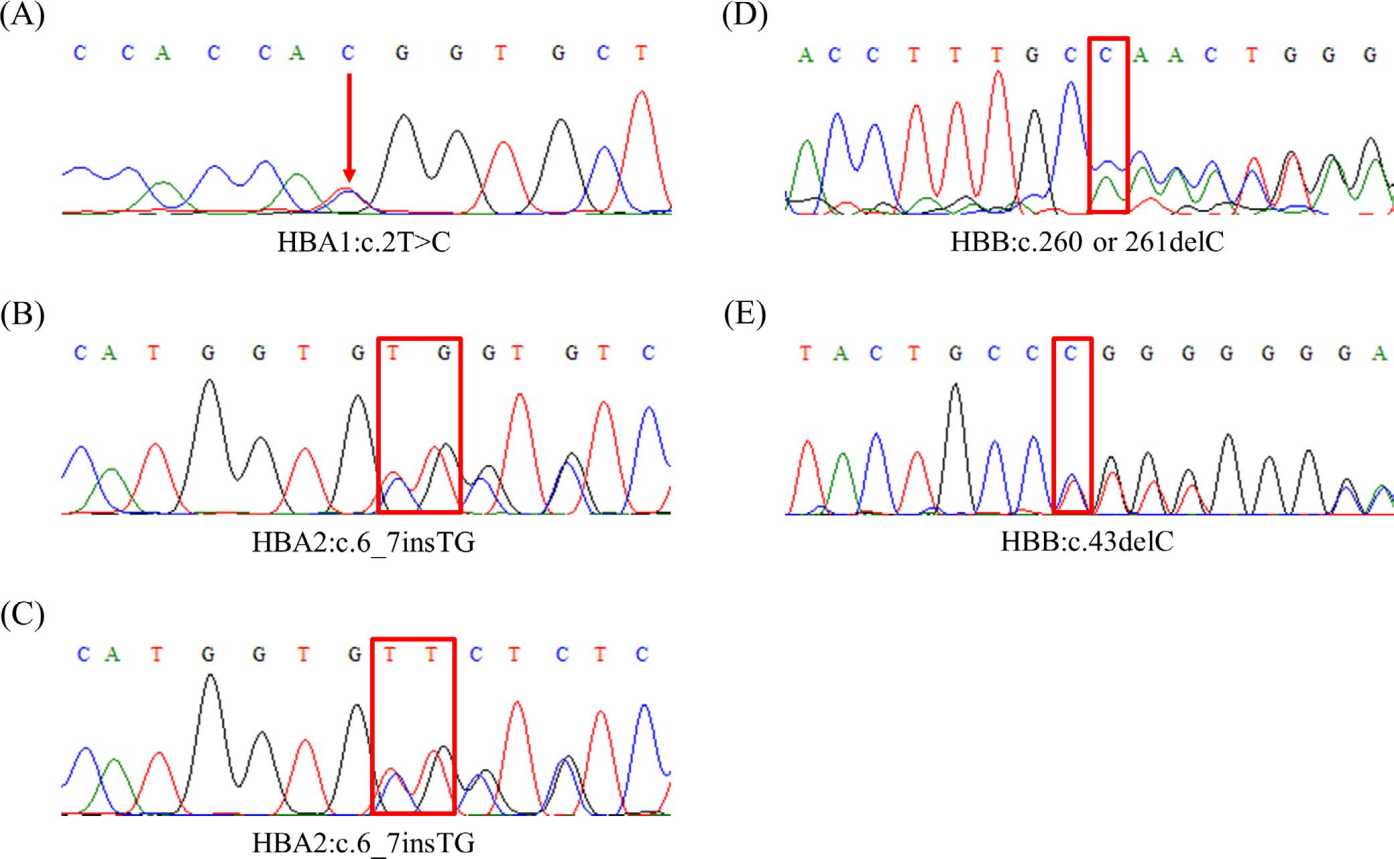
DNA sequence analysis of the probands with novel hemoglobin mutations. The point‐mutated site is labeled with red arrow, and the inserted and deleted sites are labeled with red rectangle. A, HBA1:c.2T>C in proband 1; B, HBA2:c.6_7insTG in proband 2; C, HBA2:c.6_7insTG in proband 3; D, HBB:c.260 or 261delC in proband 4; E, HBB:c.43delC in proband 5

In this study, four novel thalassemia mutations were identified by NGS in five probands and were further confirmed by Sanger sequencing (Figure 1). Among these mutations, two novel α‐thalassemia mutations have been identified in three individuals. The mutations and their hematological parameters are listed in Table 5. The mutation HBA1:c.2T>C, which was a novel mutation of the translation initiation codon of the α1‐globin gene, was found in a woman with reduced MCV and MCH. The HBA2:c.6_7insTG, resulting in a completely different polypeptide from the original alpha‐globin peptide, was observed in two unrelated individuals. They were both associated with reduced MCV and MCH. One of them was a woman who had two children. Direct DNA sequencing showed that this mutation was inherited from her mother and her two children were both heterozygous for this mutation. They all show slightly reduced MCV and MCH.

**Table 5:** Hematological data from probands with novel mutations and family members of partial probands

Case	Age (y)/sex	Genotype		Hb (g/L)	MCV (fL)	MCH (pg)	Hb A (%)	Hb A2 (%)	Hb F (%)
		α	β						
Proband 1	37/F	HBA1:c.2T>C/αα	β^N^/β^N^	104	77.2	25.7	—	—	—
Proband 2	1/M	HBA2:c.6_7insTG/αα	β^N^/β^N^	90	59.2	17.8	97.3	2.7	0
**Family A**									
Proband 3	36/F	HBA2:c.6_7insTG/αα	β^N^/β^N^	117	80.6	26	97.4	2.6	0
Mother	74/F	HBA2:c.6_7insTG/αα	β^N^/β^N^	140	83.9	26.3	97.2	2.8	0
Daughter	1/F	HBA2:c.6_7insTG/αα	β^N^/β^N^	103	70	21.6	95	2.7	2.3
Daughter	11F	HBA2:c.6_7insTG/αα	β^N^/β^N^	111	77.7	23.4	97.6	2.4	0
**Family B**									
Proband 4	22/F	αα/αα	HBB:c.260 or 261delC/β^N^	74	72.7	21.3	86.5	5.4	0
Son	1/M	αα/αα	Codons 41/42 (‐TTCT)/HBB:c.260 or 261delC	50	66.7	19.2	—	—	—
Husband	28/M	αα/αα	Codons 41/42 (‐TTCT)/β^N^	136	65.3	19.5	95.65	4.35	0
**Family C**									
Proband 5	1/F	αα/αα	HBB:c.43delC/β^N^	98	57.2	18.2	80	4.5	15.5
Father	27/M	αα/αα	HBB:c.43delC/β^N^	115	63.4	18.2	93.7	5.3	1
Mother	29/F	αα/αα	β^N^/β^N^	138	87.4	29.9	96.7	2.8	0.5

—, not available; Hb A, hemoglobin A; Hb A2, hemoglobin A2; Hb F, hemoglobin F; Hb, hemoglobin; MCH, mean corpuscular hemoglobin; MCV, mean cell hemoglobin.

Two novel β‐thalassemia mutations were discovered, and the molecular and hematological parameters of each allele are listed in Table 5. These two mutations were both frameshift mutations. The HBB:c.43delC mutation was found in a 1‐year‐old girl with significantly reduced MCV (63.4 fL) and MCH (18.2 pg). This mutation was inherited from her father, who was also associated with significantly reduced MCV (63.4 fL) and MCH (18.2 pg). The second mutation HBB:c.260 or 261del was firstly found in a pregnant woman with reduced MCV (65 fL), MCH (21.4 pg), and HBA (86.5%) and increased Hb A2 (5.4%). Her husband also carried a β0 globin mutation (Codons 41/42 (‐TTCT)). Direct sequencing of the α‐ and β‐globin genes by amniocentesis showed that the fetus was doubly heterozygous for HBB:c.260 or 261del and Codons 41/42 (‐TTCT). However, this mother insisted to keep this fetus. Now this child shows serve β‐thalassemia phenotype and needs transfusion therapy every month.

## 4 DISCUSSION

There is a high frequency of thalassemia in southern China, particularly in Guangdong, Guangxi, and Hainan provinces.^18^, ^19^ The percentage of thalassemia carriers is relatively low in most regions in Hunan Province.^21^ This disorder was neglected by provincial health system in some high‐prevalence regions due to limited molecular epidemiological data.

In this study, we report molecular epidemiological data of thalassemia in Chenzhou Region comprehensively for the first time. Our data confirmed that Chenzhou had the highest overall prevalence rate of thalassemia (10.78%) in Hunan Province, which was slightly higher than that of our previous result (10.00%).^16^ Meanwhile, the rates of α‐thalassemia and β‐thalassemia were slightly higher and less than those of our previous results, respectively. These changes were probably caused by different genetic screening methods and/or population mobility and migration. The rate of thalassemia in Chenzhou was significantly higher than the average of Hunan Province (4.18%)^22^, ^23^ and was closer to that of Guangdong Province and southern Jiangxi Province.^19^, ^24^ Notably, the rates of α and β‐thalassemia in Chenzhou had a special distribution pattern. Generally, α‐thalassemia occurs at a much higher frequency than that of β‐thalassemia; however, the rate of α‐thalassemia carriers (5.97%) was close to that of β‐thalassemia carriers (4.48%) in Chenzhou Region. This distribution pattern was consistent with the data of Changsha Region in Hunan Province and indicated that Chenzhou had a lessened rate of α‐thalassemia and a relatively higher rate of β‐thalassemia compared with surrounding provinces of southern China.^25^ Moreover, the rate of composite α and β‐thalassemia (0.34%) in Chenzhou was newly determined in this study.

In contrast to six variations and 10 genotypes identified in our previous study,^16^ we identified 30 distinct α‐thalassemia genotypes with 19 different variations here. Among α‐thalassemia genotypes, the most common subtype was ‐‐^SEA^/αα with a remarkable proportion of 67.87%. The proportion was similar to that of Changsha Region in Hunan Province and higher than that of surrounding provinces.^21^, ^25^ Apart from these common variation types, a series of rare and novel variations were identified. IVS‐I‐117 (G>A) was a rare mutation which was firstly reported in Indian population.^26^ And HBA2:c.95+5_95+28delGGCTCCCTCCCCTGCTCCGACCCG was only reported once in Malaysia.^27^ Interestingly, HBA2:c.184A>T(Lys>End) was a recently reported novel mutation which was also found by NGS in Guangdong Province, China.^28^ Furthermore, we identified two novel α‐thalassemia‐associated mutations in this study, including HBA1:c.2T>C and HBA2:c.6_7insTG. For β‐thalassemia, we identified 21 β‐thalassemia variations with 32 genotypes in this cohort, whereas only 13 mutations and 13 genotypes were identified in our previous study.^16^ Of the β‐thalassemia genotypes, Codons 41/42 (‐TTCT)/β^N^ and IVS‐II‐654 (C>T)/β^N^ were the most two frequent β‐thalassemia subtypes, accounting for 65.31% of the genotypes. And the ranking order of the two major mutations was different from our previous result.^16^ We assumed that it was probably due to population mobility and/or new genetic screening methods. In addition, a rare mutation of β‐globin gene, IVS II‐761 A>G, was firstly identified in Mainland China. IVS II‐761 A>G was previously reported once in a pan‐ethnic population of America.^29^ Furthermore, HBB:c.260 or 261delC and HBB:c.43delC were newly characterized β‐thalassemia mutations.

In this study, the prevalence of abnormal hemoglobins (0.22%) was determined in Chenzhou Region for the first time. Thirteen abnormal hemoglobin variants were identified. Among these variants, Hb Zurich‐Langstrasse, Hb Yusa, and Hb Genova were firstly reported in China. Hb Zurich‐Langstrasse and Hb Yusa were also rarely reported worldwide.^30^, ^31^ In addition, a new form of mutation (HBA2:c.150C‐>G) referred to as Hb Savaria was identified. Theoretically, Hb Savaria has three different variants, including HBA2:c.150C‐>A, HBA2:c.148A‐>C, and HBA2:c.150C‐>G. HBA2:c.150C‐>A, which is present in HbVar, was firstly reported in a female from Kenya.^32^ Another variant HBA2:c.148A‐>C was recently reported in France.^17^ The last form of mutation HBA2:c.150C‐>G was firstly found in a woman with normal hematological parameters in our study.

With the advent of NGS techniques in recent years, NGS emerged as a popular tool in prenatal screening.^33^, ^34^ So far, many recent investigations confirmed the advantage of NGS in screening thalassemia carriers and detecting novel and complex variations.^13^, ^14^, ^15^, ^36^ An investigation of prevalence and genetic background of thalassemia in Baise Region found 24 common variations and 4 rare novel variations.^13^ In another study, 49.5% of Dai people were diagnosed as thalassemia carriers via direct NGS screening. By contrast, 22.0% was found by traditional methods in the same cohort^14^. In our study, we identified 40 genomic variations including 11 rare and novel mutations by combined NGS and Gap‐PCR. Among these variations, only three types of deletion and 20 types of mutation could be detected by combined RDB and Gap‐PCR. Twenty types of genomic variation could be missed. In other words, 2.88% of all thalassemia carriers could be missed by traditional genetic analysis in this cohort. The child with compound heterozygosity of HBB:c.260 or 261del and Codons 41/42 (‐TTCT) in our study served as a prime example of the importance of identification of novel mutations in prenatal diagnosis. This result implied that the molecular background of thalassemia was much more complex than previously reported and traditional methods are not sufficient for accurate diagnosis of thalassemia. NGS could be a better mass screening method, especially in high‐prevalence regions.

Several other limitations of this study should also be considered. Although the clinical manifestations of thalassemia mainly depend on the degree of imbalance of the ratio of α:non‐α chains, individuals with identical genotypes can exhibit variable clinical severities. Genetic modulators and cis‐regulatory elements have significant effects on clinical manifestation.^5^ More loci should be covered in the NGS screening, which is important for precise diagnosis and treatment of thalassemia. Recently, a designed targeted gene panel, which included all eight globin genes and validated modulators (*KLF1*, *BCL11A*, and *MYB*), was applied in molecular screening and clinical genotyping in thalassemia. The result showed that comprehensive NGS greatly facilitates screening and diagnosis of thalassemia.^15^ However, there are still some limitations of NGS techniques. Zebisch et al^39^ identified a novel variant of epsilon‐gamma‐delta‐beta thalassemia by using MLPA and CGH, which was missed by NGS in this study. In consideration of diverse types of thalassemia variations, combined methods are needed to detect thalassemia variations completely. On the other side, new technologies such as haplotype‐resolved genome sequencing and single‐molecule real‐time sequencing may completely solve this problem in the future.^40^, ^41^

In conclusion, we demonstrated the great diversity of thalassemia‐associated variations and a high prevalence of thalassemia in Chenzhou Region by applying combined NGS and Gap‐PCR technology. The rare and novel variations of this region were identified for the first time. Our findings would be meaningful for prevention and treatment of thalassemia in this region and other high‐prevalence areas. Our updated epidemiological data may also draw the attention of local governments in the severity of this disorder, and more public funds might be allocated for prevention. Limitations of our study, such as possible missed carriers during primary screening and complex variations, will be further addressed in the future.

## CONFLICT OF INTEREST

The authors declare no conflict of interest.
